# Publicly Available Large Language Models for Trichoscopy: A Head-to-Head Comparison with Dermatologists

**DOI:** 10.3390/diagnostics16010169

**Published:** 2026-01-05

**Authors:** Basil Signer, Ali Mokhtari, Simone Cazzaniga, Flurin Brand, Gemma Caro, Pierre A. de Viragh, Kristine Heidemeyer, Aref Hosseini, Matilde Iorizzo, Alexandra Junge, Zora Martignoni, Pascal Edouard Reygagne, Bianca Maria Piraccini, Michela Starace, Antonia Reimer-Taschenbrecker, Charlotte Vogel, Dominik Obrist, Seyed Morteza Seyed Jafari

**Affiliations:** 1 Department of Dermatology, Inselspital, Bern University Hospital, 3010 Bern, Switzerland; 2 ARTORG Center for Biomedical Engineering Research, University of Bern, 3010 Bern, Switzerland; 3 Department of Dermatology, Ente Ospedaliero Cantonale (EOC), Bellinzona Regional Hospital, 6500 Bellinzona, Switzerland; 4 Department of Dermatology, University Teaching and Research Hospital of the University of Lucerne, 6000 Lucerne, Switzerland; 5 Private Dermatology Practice, 6500 Bellinzona, Switzerland; 6 Centre Sabouraud, Hôpital St. Louis, 75010 Paris, France; 7 Private Dermatology Practice, 40137 Bologna, Italy; 8 Dermatology Unit, IRCCS Azienda Ospedaliero-Universitaria di Bologna, 40138 Bologna, Italy; 9 Department of Medical and Surgical Sciences, Alma Mater Studiorum University of Bologna, 40126 Bologna, Italy

**Keywords:** alopecia, artificial intelligence, comparative study, dermatologists, diagnostic accuracy, large language models, prospective study, trichoscopy

## Abstract

**Background/Objectives:** Trichoscopy is an important diagnostic tool for hair and scalp disorders, but it requires significant expertise. Publicly available large language models (LLMs) are becoming more popular among both physicians and patients, yet their usefulness in trichology is unknown. We aimed to evaluate the diagnostic accuracy of four publicly available LLMs when interpreting trichoscopic images, as well as to compare their performance with that of dermatology residents, board-certified dermatologists, and trichology experts. **Method:** In this prospective comparative study, a preprocessed set of trichoscopic images was assessed in an online image-based survey. To reduced recognition bias from public image repositories, all images were structurally transformed while preserving diagnostic features. Fifteen dermatologists (five residents, four board-certified dermatologists, six trichology experts) provided a suspected diagnosis (SD), and up to three the differential diagnoses (DD). Four LLMs (ChatGPT-4o, Claude Sonnet 4, Gemini 2.5 Flash, and Grok-3) evaluated the images under the same conditions. **Results:** The overall diagnostic accuracy among 15 dermatologists was 58.1% (95% CI, 53.0–63.0) for SD and 68.3% (95% CI, 63.4–72.8) for SD + DD. Experts significantly outperformed residents and board-certified dermatologists. AI models achieved an accuracy of 18.2% (95% CI, 11.8–26.9) for SD and 44.4% (95% CI, 35.0–54.3) for SD + DD. Gemini 2.5 Flash performed best, with an accuracy of 62.5% for SD + DD. Agreement among dermatologists increased with experience (AC1 up to 0.65 for experts), while agreement among AI models was moderate to good (AC1 up to 0.70). Agreement between AI models and dermatologists was only slight to fair (AC1 = 0.06 for SD and 0.21 for SD + DD). All human-AI differences were statistically significant (*p* < 0.001). **Conclusions:** In trichology, publicly available LLMs currently underperform compared to human experts, especially in providing a single correct diagnosis. These models require further development and specialized training before they can reliably assist with trichological diagnoses in routine care.

## 1. Introduction

Dermoscopy is a noninvasive technique that employs a light source and a magnifying lens to enable close examination of the skin. It illuminates colors, patterns, and structures beneath the surface that are not seen macroscopically, offering a clearer view of the epidermis and upper layer of the dermis [1]. When this method is applied to the scalp, it is referred to as trichoscopy. Trichoscopy has become an important tool for diagnosing hair and scalp conditions and is now one of the main applications of dermoscopy, second only to its use in diagnosing skin cancer, demonstrating high diagnostic accuracy [2]. However, the method relies on clinical experience, which may lead to misdiagnoses due to clinical diversity and overlapping trichoscopy patterns [3].

The use of artificial intelligence (AI) is increasing rapidly, publicly available Large Language Models (LLMs) such as ChatGPT receive over 10 million queries daily, of which an estimated 5–10% relate to medical or health topics [4]. This trend underscores the growing importance of AI in healthcare research, clinical decision-making and potential patient education [5,6]. LLMs represent a major advance in natural language processing (NLP), capable of generating human-like text based on massive datasets and reinforcement learning from human feedback. Traditional machine learning and deep learning models focus primarily on analysis whereas LLMs are capable of processing and interpreting text inputs [7]. Furthermore, their use is expanding beyond text processing to include image analysis [6]. Therefore, the AI-assisted smartphone apps and web-based services for skin disease represent the next logical step, as trichoscopy is a visual field based on pattern recognition. According to the position statement of the EADV Artificial Intelligence Task Force, AI has the potential to greatly benefit patients by improving access to diagnosis, treatment, and education [8], particularly given the notable shortage and uneven distribution of dermatologists, which is pronounced in remote areas [9]. In trichology, deep learning systems are rapidly evolving, promising fast and effective support for the diagnosis and treatment of hair disorders [10]. A recent study indicates that a specifically tailored AI model can achieve diagnostic accuracy for scalp and trichological conditions that is comparable to, and in some cases exceeds, that of human experts with a reported diagnostic accuracy of 92% [10]. However, the availability of such specially trained models is often limited to research settings, trichology clinics, and specialists, and they are not yet integrated into daily clinical practice. Today, patients may present the physician with an AI-suspected diagnosis and expect certain clinical evaluation or treatment steps accordingly, which changes the physician-patient relationship [11].

In this prospective comparative study, we aimed to analyze the diagnostic accuracy of four publicly available LLMs, which did not have specific training to recognize scalp and hair disorders. The results of their assessment were compared to that of residents in dermatology, board-certified dermatologists, and experts in trichology.

## 2. Materials and Methods

**Study Design:** We performed a prospective, comparative study analyzing the capabilities of AI in a manner relevant to everyday clinical and consumer use. We selected four well-known and easily accessible multimodal AI models: ChatGPT-4o (OpenAI), Claude Sonnet 4 (Anthropic), Gemini 2.5 Flash (Google DeepMind), and Grok-3 (xAI). These models represent some of the most advanced general-purpose AI systems currently available and are accessible through widely used platforms, making them representative of the tools that healthcare professionals or laypersons might realistically use. All four models support image input and text-based reasoning, making them suitable for tasks involving visual diagnosis and explanation. By testing these systems with our preprocessed image dataset, we aimed to assess their diagnostic performance under conditions where prior exposure to the exact images would be improbable. Furthermore, the systems were not prompted. This enabled us to more accurately characterize the real-world generalizability of these models when applied to dermatological cases.

We recruited dermatology residents, board-certified dermatologists, and trichology experts as a control group. In an online questionnaire, the participants were tasked with providing a primary diagnosis and potentially three differential diagnoses after seeing a trichoscopic image. Additionally, they were asked to provide a recommendation for further diagnostic steps and, if applicable, treatment approach. No information was provided about the patient’s status or medical history.

**Data Preparation:** A central methodological concern in evaluating AI diagnostic systems is the risk of dataset leakage and recognition bias due to the overlap between test images and training data, particularly in models relying on large-scale pretraining. Likely many dermatological images in public datasets have been incorporated, directly or indirectly, into the training corpora of advanced AI systems. Using such images in unaltered form risks overestimating the efficiency of AI due to duplicate or near-duplicate recognition, thereby compromising the scientific validity of comparative evaluations against clinical experts.

To mitigate this risk, we developed a structured image-preprocessing pipeline that renders test images unrecognizable to pretrained AI models while preserving clinically relevant features essential for human diagnosis. This pipeline, implemented in Python (3.12.7) using OpenCV (4.11.0), NumPy (1.26.4), PIL (10.4.0), SciPy (1.13.1), and PyTorch (2.9.0), applied a reproducible sequence of transformations to all images.

Initial steps included minor cropping (0.75% per side) to eliminate border artifacts followed by the addition of uniform white padding (1.5%) to subtly modify image composition. Geometric transformations were applied next, including random scaling (between 90% and 110% of the original size) and constrained rotations (−3 to +3 degrees). These adjustments were designed to emulate plausible photographic variation while preserving the structural integrity of dermatological lesions, ensuring that diagnostic morphology remained unaffected.

Photometric changes were then introduced to further diversify image appearance across lighting and color conditions. These changes included minor random adjustments to brightness and contrast (within the range of 1.01 to 1.04), modest increases in saturation (1.005 to 1.01), and gamma correction with values ranging from 0.8 to 1.2. Such transformations were intentionally subtle, designed to reflect realistic variations in acquisition environments while maintaining diagnostic clarity and lesion visibility. To disrupt low-level feature memorization, we added light Gaussian blur (kernel size 3), low-variance Gaussian noise, and a controlled perturbation based on smoothed random noise.

All transformed images were saved separately to preserve the original data and ensure full reproducibility. Their diagnostic validity was independently confirmed by two board-certified dermatologists, ensuring that the applied modifications maintained clinical interpretability and did not compromise the integrity of the human–AI performance comparison.

**Statistical analysis:** We presented categorical variables as absolute numbers and percentages for the descriptive analysis. To evaluate the performance of both participants and AI algorithms, each response was compared against a true, verified diagnosis for each case. A response was considered correct if the suspected diagnosis (SD) alone or the combination of the suspected diagnosis and differential diagnosis (SD + DD) included the true diagnosis.

To determine the diagnostic accuracy, we calculated the percentage of correct responses relative to the total number of evaluations and cases, presenting results with 95% confidence intervals (CI). We used Pearson’s Χ^2^ test or Fisher’s exact test, when appropriate, to compare the accuracy between groups. If a significant difference was found, we applied the Holm-Bonferroni method for post hoc subgroup comparisons.

We measured the inter-rater reliability, defined as the agreement among evaluators in answering the same cases correctly or incorrectly, using Gwet’s AC1 statistic with 95% CI [12]. This statistic is interpreted similarly to Cohen’s kappa and can be categorized as follows: <0 poor, 0–0.20 slight, 0.21–0.40 fair, 0.41–0.60 moderate, 0.61–0.80 good, and 0.81–1 very good agreement. All tests were considered statistically significant at *p*-value < 0.05. Statistical analyses were performed using R software version 4.3.3 (R Foundation for Statistical Computing, Vienna, Austria).

## 3. Results

Overall, 15 dermatologists took part in the study: 5 residents (33.3%), 4 board-certified dermatologists (26.7%), and 6 (40.0%) experts in trichology (Table 1).

**Figure 1:**
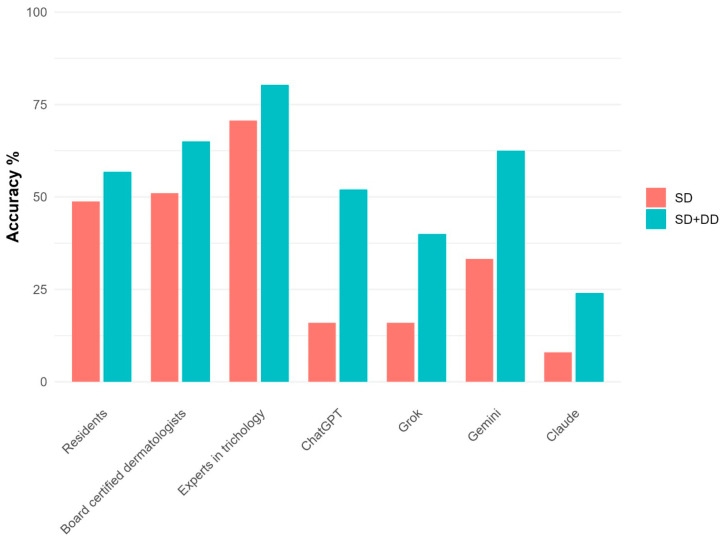
Diagnostic accuracy for each group of evaluators in detail.

The overall diagnostic accuracy of dermatologists was 58.1% (95% CI: 53.0, 63.0) and 68.3% (63.4, 72.8) of the cases for SD and SD + DD respectively (Table 2). By contrast, AI accuracy was 18.2% (11.8, 26.9) for SD and 44.4% (35.0, 54.3) for SD + DD. These differences were all statistically significant (*p* < 0.001, Supplementary Table S1). When evaluating the accuracy across dermatologist groups, a clear upward trend was observed ranging from residents (56.8% for SD + DD) to trichology experts (80.3%) (Figure 1). Statistically significant differences were observed between residents and experts, as well as between board-certified dermatologists and experts. Regarding AI, the accuracy of algorithms was comparable for SD and significantly different for SD + DD (*p* = 0.04), with Gemini showing a better performance (62.5% for SD + DD). However, the specific post hoc comparisons between AI algorithms did not reveal any statistically significant difference. Diagnostic accuracy for each case, in total and by group of evaluators, is reported in Supplementary Table S2.

Table 3 shows the inter-rater reliability, in total and by group of evaluators. The overall agreement among dermatologists, as measured by AC1 statistic, was 0.41 (0.23, 0.59) for SD and 0.44 (0.22, 0.66) for SD + DD. Reliability rose with increasing clinical experience, ranging from 0.43 for residents (SD + DD) to 0.65 for experts, with some heterogeneity between groups. AI algorithms showed a good level of mutual agreement for SD, with AC1 = 0.70 (0.50, 0.90), and a moderate agreement for SD + DD, with AC1 = 0.41 (0.18, 0.63). However, the agreement between dermatologists and AI was only slight for SD (AC1 = 0.06) and fair for SD + DD (AC1 = 0.21).

## 4. Discussion

The use of AI for assessing scalp and hair diseases is currently exclusive to research settings. Several deep learning models have demonstrated in controlled studies that their diagnostic accuracy matches or exceeds that of human experts. Recent work using advanced convolutional neural networks, such as modified Xception and multi-network fusion object detection frameworks, has shown diagnostic accuracies of up to 92% for a range of hair and scalp disorders, and superior performance compared to dermatologists in distinguishing conditions like scalp psoriasis from seborrheic dermatitis [3,13]. Other systems have been used to facilitate the diagnosis of alopecia areata with a computer based system to reproduce the Severity of Alopecia Tool (SALT) [14,15]. In another study a tool was used to classify 675 images of men in corresponding stages of male pattern baldness, archiving average accuracy of 82–86% [16]. Further an imaging devices for diagnosis of scalp conditions using AI were proposed, one tested system achieved 97–99% accuracy in detecting dandruff, folliculitis, hair loss and oily hair [17,18]. According to Gupta et al., deep learning databases for the classification of scalp dermoscopy images will require significant and collaborative efforts from hair specialists, but they could become valuable diagnostic for both physicians and patients. Currently, their role is limited to research and supplementary support until further validation and regulatory approvals are obtained [10].

By contrast, publicly available LLMs (like ChatGPT) are already widely used by physicians and patients and have demonstrated advanced skills, such as passing the United States Medical Licensing Examination and outperforming 99.98% simulated humans in diagnosing complex medical cases [19,20]. However, their accuracy remains unverified in highly specialized fields as trichology.

In this study, dermatologists achieved a mean accuracy of 58.1% when limited to the suspected diagnosis and 68.3% when including differential diagnoses. Experts in trichology outperformed both dermatology residents and board-certified dermatologists, confirming the difficulty in evaluating hair and scalp disorders. The tested AI models showed significantly lower diagnostic accuracy and were outperformed by all three dermatology. They showed limited diagnostic value by correctly identifying the suspected diagnosis in only 18.2% of cases. When differential diagnoses were included, AI reached 44.4% accuracy. Notably, AI failed to identify the correct diagnosis in several cases that were correctly identified by most dermatologists (e.g., case 1, benign nevus—Supplementary Materials) and achieved perfect accuracy only in one case (case 16, melanoma). Interestingly, Gemini was the best-performing AI (62.5% for SD + DD). The comparison across AI models raises questions about differences in training data and architecture among publicly available models.

In cases where dermatologists performed highly but AI underperformed (e.g., case 6, hemangioma or case 1, benign nevus), most involved images of benign tumors. However, AI models consistently misclassified these as malignant lesions, unscoring the previously reported difficulty of LLMs in differentiating between benign and malignant lesions [21]. AI performed comparatively better in a few cases, mostly regarding relatively common inflammatory diseases. Previous studies and systematic reviews reported similar findings, observing high accuracy for common skin diseases and lower recognition rates of rare conditions and lower represented skin types [22]. AI models require a large and diverse dataset in order to achieve a high diagnostic accuracy. Common diseases are overrepresented in clinical image repositories leading to better training but further generalization. Additionally, rare diseases may be underrepresented, limiting the ability of models to distinguish them [23,24]. These findings were supported by our study when examining cases that posed significant challenges for both AI and dermatologists, where AI achieved the lowest accuracy (e.g., case 9, central centrifugal cicatricial alopecia or case 17, hair dye). These results support recent findings, that showed that publicly available LLMs struggle in the field of trichoscopy [25].

Trichology experts had the highest inter-rater reliability, indicating both better accuracy and more consensus. High variability in agreement between AI and dermatologists suggests that AI often “thinks differently,” perhaps relying more on pattern matching. This low concordance raises concerns about how clinicians could (or should) interpret AI recommendations, especially if they contradict human assessment.

This study has several limitations. First, the relatively small sample size (human participants and LLMs) does not allow a generalization.

Second, we would like to highlight the difficulty of providing a diagnosis only providing a clinical image that does not reflect the complexity of a real-world setting, whereases physician are provided with a patient history, examine the entire skin, evaluate the growth pattern and can exam different parts of the scalp with trichoscopy in combination with histopathological correlation. Additionally, the consecutive analysis of the cases may have caused physician fatigue. Thus, these may have performed better in a more real-life situation. On the other side the LLMs were not prompted and the used systems are not specifically trained for diagnosis of scalp disease, which may have lowered accuracy values of them.

Further, the use of publicly available images introduces the possibility that some cases may be recognizable or inferable by LLMs. To address this, we developed a structured and reproducible image pre-processing pipeline to create a diagnostically valid dataset that is clearly distinct from publicly accessible sources. This limitation further reflects a broader, unresolved ethical issue concerning the use of patient images in the training and deployment of LLM-based platforms, an area that warrants ongoing ethical and technical discussion. In addition, trichoscopy usually requires a dermatoscope and camera adapter, limiting patient use. However, newer smartphone cameras (e.g., iPhone Macro Mode) may provide sufficient quality for at-home assessments, potentially supporting AI-based evaluation for initial monitoring or follow-up.

## 5. Conclusions

Although AI models show promise in medical applications, their current diagnostic performance in trichology is inferior to that of dermatologists at all levels of education. While current AI models underperform in this area, future models that are specifically trained on trichology cases could support clinical decision-making and potentially replace clinical and physical examinations. Until then, specialist evaluation is essential for accurate diagnosis in this complex field.

## Figures and Tables

**Table 1:** Characteristics of dermatologists included in the study.

	*N* = 15 (%)
Training status	
Residents	5 (33.3)
Board-certified dermatologists	4 (26.7)
Experts in trichology	6 (40.0)
Experience	
1–5 years	5 (33.3)
6–10 years	3 (20.0)
11–20 years	2 (13.3)
21+ years	5 (33.3)
Working place	
University hospital	7 (46.7)
Public hospital	3 (20.0)
Private hospital	1 (6.7)
Private practice	4 (26.7)

Due to rounding, percentages may not add up to 100%.

**Table 2:** Diagnostic accuracy of each group of evaluators, in total and in detail.

Group	SD % (95% CI)	SD + DD % (95% CI)
Dermatologists	58.1 (53.0, 63.0)	68.3 (63.4, 72.8)
Residents	48.8 (40.2, 57.5)	56.8 (48.0, 65.2)
Board-certified dermatologists	51.0 (41.3, 60.6)	65.0 (55.3, 73.6)
Experts in trichology	70.7 (62.9, 77.5)	80.3 (73.1, 85.9)
AI	18.2 (11.8, 26.9)	44.4 (35.0, 54.3)
ChatGPT	16.0 (6.4, 34.7)	52.0 (33.5, 70.0)
Grok	16.0 (6.4, 34.7)	40.0 (23.4, 59.3)
Gemini	33.3 (18.0, 53.3)	62.5 (42.7, 78.8)
Claude	8.0 (2.2, 25.0)	24.0 (11.5, 43.4)

AI: artificial intelligence algorithms, CI: confidence interval, DD: differential diagnosis, SD: suspected diagnosis.

**Table 3:** Inter-rater reliability, in total and by group of evaluators.

Group	SD AC1 (95% CI)	SD + DD AC1 (95% CI)
Dermatologists	0.41 (0.23, 0.59)	0.44 (0.22, 0.66)
Residents	0.47 (0.27, 0.68)	0.43 (0.21, 0.66)
Board-certified dermatologists	0.35 (0.12, 0.58)	0.45 (0.19, 0.71)
Experts in trichology	0.56 (0.31, 0.81)	0.65 (0.44, 0.85)
Between groups	0.48 (0.19, 0.77)	0.52 (0.21, 0.82)
AI	0.70 (0.50, 0.90)	0.41 (0.18, 0.63)
Dermatologists vs. AI	0.06 (−0.42, 0.53)	0.21 (−0.21, 0.63)

AI: artificial intelligence algorithms, AC1: Gwet’s measure of agreement for binary ratings, CI: confidence interval, DD: differential diagnosis, SD: suspected diagnosis.

## Data Availability

The data supporting the results of this study are presented in the current paper.

## Supplementary Materials

The following supporting information can be downloaded at https://www.mdpi.com/article/10.3390/diagnostics16010169/s1, Study Questionnaire, Study Cases, Table S1: Comparisons of diagnostic accuracy by group of evaluators, Table S2: Diagnostic accuracy for each case, in total and by group of evaluators.

## Abbreviations

The following abbreviations are used in this manuscript:

AI	Artificial intelligence
LLMs	large language models
