# Supplementary material for: Publicly Available Large Language Models for Trichoscopy: A Head-to-Head Comparison with Dermatologists
Source: Diagnostics (Basel). 2026 Jan 5;16(1):169. doi: 10.3390/diagnostics16010169 (PMC12785308; doi:10.3390/diagnostics16010169)
Supplement: Supplementary file 1 [file diagnostics-16-00169-s001.zip › diagnostics-3999386-supplementary.pdf]

Supplementary Material

Study Questionnaire

Following questions were the physicians and AI asked only showing 25 different dermatoscopy/trichoscopy images of the scalps disease:

1) What is your primary suspected diagnosis?
2) Please name up to three other differential diagnoses, if any.
3) What diagnostic step(s), if any, would you recommend?
4) What primary treatment, if any, would you recommend?

Supplementary Material

Study Cases

The following diagnoses were included in the patient images shown:

1) Benign nevus of the scalp
2) Tinea capitis
3) Basal cell carcinoma of the scalp
4) Scalp Psoriasis
5) Blue nevus of the scalp
6) Hemangioma of the scalp
7) Discoid lupus erythematosus
8) Frontal fibrosing alopecia
9) Central centrifugal cicatricial alopecia
10) Lichen planopilaris
11) Trichotillomanie
12) Alopecia areata
13) Telogen effluvium
14) Androgenetic alopecia
15) Dissecting cellulitis of the clap
16) Melanoma
17) Hair dye
18) Pyogenic granuloma of the scalp
19) Nevus sebaceous
20) Seborrheic Dermatitis
21) Folliculitis decalvans
22) Cherry angioma of the scalp
23) Trichorrhexis invaginata
24) Monilethrix
25) Seborrheic keratosis of the scalp

Suppl. Table S1 - Comparisons of diagnostic accuracy by group of evaluators

Group	SD	SD+DD
	P*	P*
Dermatologists (among all subgroups)	<0.001	<0.001
Residents vs Board certified dermatologists	0.85	0.27
Residents vs Experts in trichology	0.001	<0.001
Board certified dermatologists vs Experts in trichology	0.005	0.02
Dermatologists vs AI	<0.001	<0.001
AI (among all algorithms)	0.17	0.04
ChatGPT vs Grok	-	>0.99
ChatGPT vs Gemini	-	>0.99
ChatGPT vs Claude	-	0.40
Grok vs Gemini	-	0.79
Grok vs Claude	-	>0.99
Gemini vs Claude	-	0.09

AI: artificial intelligence algorithms, DD: differential diagnosis, SD: suspected diagnosis

* Pearson's X^2 test or Fisher's exact test if required. For subgroup comparisons p-values were adjusted by Holm–Bonferroni method.

Suppl. Table S2 - Diagnostic accuracy for each case, in total and by group of evaluators

Case	Total Dermatologists (N = 15)		Residents (N = 5)		Board certified dermatologists (N = 4)		Experts in trichology (N = 6)		AI (N = 4)	
	SD	SD+DD	SD	SD+DD	SD	SD+DD	SD	SD+DD	SD	SD+DD
1	100 (79.6, 100)	100 (79.6, 100)	100 (56.6, 100)	100 (56.6, 100)	100 (51.0, 100)	100 (51.0, 100)	100 (61.0, 100)	100 (61.0, 100)	0.0 (0.0, 49.0)	25.0 (4.6, 69.9)
2	73.3 (48.0, 89.1)	73.3 (48.0, 89.1)	40.0 (11.8, 76.9)	40.0 (11.8, 76.9)	75.0 (30.1, 95.4)	75.0 (30.1, 95.4)	100 (61.0, 100)	100 (61.0, 100)	25.0 (4.6, 69.9)	75.0 (30.1, 95.4)
3	93.3 (70.2, 98.8)	93.3 (70.2, 98.8)	100 (56.6, 100)	100 (56.6, 100)	75.0 (30.1, 95.4)	75.0 (30.1, 95.4)	100 (61.0, 100)	100 (61.0, 100)	0.0 (0.0, 56.1)	100 (43.9, 100)
4	13.3 (3.7, 37.9)	33.3 (15.2, 58.3)	0.0 (0.0, 43.4)	0.0 (0.0, 43.4)	0.0 (0.0, 49.0)	25.0 (4.6, 69.9)	33.3 (9.7, 70.0)	66.7 (30.0, 90.3)	0.0 (0.0, 49.0)	50.0 (15.0, 85.0)
5	6.7 (1.2, 29.8)	60.0 (35.7, 80.2)	0.0 (0.0, 43.4)	60.0 (23.1, 88.2)	25.0 (4.6, 69.9)	100 (51.0, 100)	0.0 (0.0, 39.0)	33.3 (9.7, 70.0)	25.0 (4.6, 69.9)	25.0 (4.6, 69.9)
6	86.7 (62.1, 96.3)	100 (79.6, 100)	100 (56.6, 100)	100 (56.6, 100)	75.0 (30.1, 95.4)	100 (51.0, 100)	83.3 (43.6, 97.0)	100 (61.0, 100)	0.0 (0.0, 49.0)	0.0 (0.0, 49.0)
7	53.3 (30.1, 75.2)	53.3 (30.1, 75.2)	20.0 (3.6, 62.4)	20.0 (3.6, 62.4)	25.0 (4.6, 69.9)	25.0 (4.6, 69.9)	100 (61.0, 100)	100 (61.0, 100)	0.0 (0.0, 49.0)	25.0 (4.6, 69.9)

Case	Total Dermatologists (N = 15)		Residents (N = 5)		Board certified dermatologists (N = 4)		Experts in trichology (N = 6)		AI (N = 4)	
	SD	SD+DD	SD	SD+DD	SD	SD+DD	SD	SD+DD	SD	SD+DD
8	33.3 (15.2, 58.3)	33.3 (15.2, 58.3)	20.0 (3.6, 62.4)	20.0 (3.6, 62.4)	25.0 (4.6, 69.9)	25.0 (4.6, 69.9)	50.0 (18.8, 81.2)	50.0 (18.8, 81.2)	0.0 (0.0, 49.0)	0.0 (0.0, 49.0)
9	13.3 (3.7, 37.9)	26.7 (10.9, 52.0)	0.0 (0.0, 43.4)	0.0 (0.0, 43.4)	0.0 (0.0, 49.0)	0.0 (0.0, 49.0)	33.3 (9.7, 70.0)	66.7 (30.0, 90.3)	0.0 (0.0, 49.0)	25.0 (4.6, 69.9)
10	86.7 (62.1, 96.3)	86.7 (62.1, 96.3)	60.0 (23.1, 88.2)	60.0 (23.1, 88.2)	100 (51.0, 100)	100 (51.0, 100)	100 (61.0, 100)	100 (61.0, 100)	25.0 (4.6, 69.9)	25.0 (4.6, 69.9)
11	46.7 (24.8, 69.9)	66.7 (41.7, 84.8)	20.0 (3.6, 62.4)	40.0 (11.8, 76.9)	25.0 (4.6, 69.9)	75.0 (30.1, 95.4)	83.3 (43.6, 97.0)	83.3 (43.6, 97.0)	25.0 (4.6, 69.9)	75.0 (30.1, 95.4)
12	92.9 (68.5, 98.7)	92.9 (68.5, 98.7)	80.0 (37.6, 96.4)	80.0 (37.6, 96.4)	100 (51.0, 100)	100 (51.0, 100)	100 (56.6, 100)	100 (56.6, 100)	25.0 (4.6, 69.9)	75.0 (30.1, 95.4)
13	13.3 (3.7, 37.9)	53.3 (30.1, 75.2)	0.0 (0.0, 43.4)	40.0 (11.8, 76.9)	0.0 (0.0, 49.0)	25.0 (4.6, 69.9)	33.3 (9.7, 70.0)	83.3 (43.6, 97.0)	0.0 (0.0, 49.0)	25.0 (4.6, 69.9)
14	46.7 (24.8, 69.9)	66.7 (41.7, 84.8)	40.0 (11.8, 76.9)	80.0 (37.6, 96.4)	50.0 (15.0, 85.0)	50.0 (15.0, 85.0)	50.0 (18.8, 81.2)	66.7 (30.0, 90.3)	0.0 (0.0, 49.0)	75.0 (30.1, 95.4)
15	26.7 (10.9, 52.0)	33.3 (15.2, 58.3)	0.0 (0.0, 43.4)	0.0 (0.0, 43.4)	25.0 (4.6, 69.9)	25.0 (4.6, 69.9)	50.0 (18.8, 81.2)	66.7 (30.0, 90.3)	0.0 (0.0, 49.0)	50.0 (15.0, 85.0)

Case	Total Dermatologists (N = 15)		Residents (N = 5)		Board certified dermatologists (N = 4)		Experts in trichology (N = 6)		AI (N = 4)	
	SD	SD+DD	SD	SD+DD	SD	SD+DD	SD	SD+DD	SD	SD+DD
16	100 (79.6, 100)	100 (79.6, 100)	100 (56.6, 100)	100 (56.6, 100)	100 (51.0, 100)	100 (51.0, 100)	100 (61.0, 100)	100 (61.0, 100)	100 (51.0, 100)	100 (51.0, 100)
17	13.3 (3.7, 37.9)	13.3 (3.7, 37.9)	20.0 (3.6, 62.4)	20.0 (3.6, 62.4)	0.0 (0.0, 49.0)	0.0 (0.0, 49.0)	16.7 (3.0, 56.4)	16.7 (3.0, 56.4)	0.0 (0.0, 49.0)	0.0 (0.0, 49.0)
18	60.0 (35.7, 80.2)	73.3 (48.0, 89.1)	60.0 (23.1, 88.2)	80.0 (37.6, 96.4)	50.0 (15.0, 85.0)	50.0 (15.0, 85.0)	66.7 (30.0, 90.3)	83.3 (43.6, 97.0)	0.0 (0.0, 49.0)	0.0 (0.0, 49.0)
19	33.3 (15.2, 58.3)	46.7 (24.8, 69.9)	20.0 (3.6, 62.4)	20.0 (3.6, 62.4)	25.0 (4.6, 69.9)	75.0 (30.1, 95.4)	50.0 (18.8, 81.2)	50.0 (18.8, 81.2)	0.0 (0.0, 49.0)	0.0 (0.0, 49.0)
20	53.3 (30.1, 75.2)	73.3 (48.0, 89.1)	80.0 (37.6, 96.4)	80.0 (37.6, 96.4)	25.0 (4.6, 69.9)	75.0 (30.1, 95.4)	50.0 (18.8, 81.2)	66.7 (30.0, 90.3)	75.0 (30.1, 95.4)	100 (51.0, 100)
21	93.3 (70.2, 98.8)	100 (79.6, 100)	100 (56.6, 100)	100 (56.6, 100)	75.0 (30.1, 95.4)	100 (51.0, 100)	100 (61.0, 100)	100 (61.0, 100)	25.0 (4.6, 69.9)	25.0 (4.6, 69.9)
22	100 (79.6, 100)	100 (79.6, 100)	100 (56.6, 100)	100 (56.6, 100)	100 (51.0, 100)	100 (51.0, 100)	100 (61.0, 100)	100 (61.0, 100)	50.0 (15.0, 85.0)	50.0 (15.0, 85.0)
23	66.7 (41.7, 84.8)	73.3 (48.0, 89.1)	60.0 (23.1, 88.2)	60.0 (23.1, 88.2)	50.0 (15.0, 85.0)	75.0 (30.1, 95.4)	83.3 (43.6, 97.0)	83.3 (43.6, 97.0)	0.0 (0.0, 49.0)	0.0 (0.0, 49.0)

Case	Total Dermatologists (N = 15)		Residents (N = 5)		Board certified dermatologists (N = 4)		Experts in trichology (N = 6)		AI (N = 4)	
	**SD**	**SD+DD**	**SD**	**SD+DD**	**SD**	**SD+DD**	**SD**	**SD+DD**	**SD**	**SD+DD**
24	57.1 (32.6, 78.6)	57.1 (32.6, 78.6)	20.0 (3.6, 62.4)	20.0 (3.6, 62.4)	50.0 (15.0, 85.0)	50.0 (15.0, 85.0)	100 (56.6, 100)	100 (56.6, 100)	50.0 (15.0, 85.0)	100 (51.0, 100)
25	92.9 (68.5, 98.7)	100 (78.5, 100)	80.0 (37.6, 96.4)	100 (56.6, 100)	100 (51.0, 100)	100 (51.0, 100)	100 (56.6, 100)	100 (56.6, 100)	25.0 (4.6, 69.9)	100 (51.0, 100)
Total	*58.1 (53.0, 63.0)*	*68.3 (63.4, 72.8)*	*48.8 (40.2, 57.5)*	*56.8 (48.0, 65.2)*	*51.0 (41.3, 60.6)*	*65.0 (55.3, 73.6)*	*70.7 (62.9, 77.5)*	*80.3 (73.1, 85.9)*	*18.2 (11.8, 26.9)*	*44.4 (35.0, 54.3)*

AI: artificial intelligence algorithms, DD: differential diagnosis, SD: suspected diagnosis

Results are presented as accuracy % with 95% confidence intervals in brackets
